# Clinical Case and Follow‐Up of *Babesia banethi* Infection in a Red Fox

**DOI:** 10.1002/vms3.71184

**Published:** 2026-08-27

**Authors:** Mariaelisa Carbonara, Michela Prioletti, Antonio Camarda, Stefano Castellana, Giuseppe Menga, Giovanna Girardi, Gad Baneth, Domenico Otranto

**Affiliations:** ^1^ Department of Veterinary Medicine University of Bari Valenzano Italy; ^2^ Istituto Zooprofilattico Sperimentale della Puglia e della Basilicata Foggia Italy; ^3^ Veterinary Diagnostic Laboratory ACV Triggiano Triggiano Italy; ^4^ Koret School of Veterinary Medicine The Hebrew University Rehovot Israel; ^5^ Department of Veterinary Clinical Sciences City University of Hong Kong Hong Kong Hong Kong; ^6^ Chulalongkorn University Bangkok Thailand

**Keywords:** *Babesia banethi*, diagnosis, longitudinal case, red foxes, therapy

## Abstract

**Background:**

Red foxes play an important role in the circulation of ticks and tick‐borne pathogens, including *Babesia* spp. While for *Babesia vulpes* many studies are available on its epidemiology in fox populations as well as pathogenicity in dogs, no data are reported for the recently described *Babesia banethi* infecting red foxes.

**Objective:**

Here we describe the clinical and parasitological follow‐up of a naturally occurring *B. banethi* infection in a red fox from southern Italy.

**Case presentation:**

An adult male fox presented with severe debilitation, weight loss, pale mucous membranes, brown discolouration of urine and sarcoptic mange. Haematological examination revealed regenerative anaemia associated with left‐shift neutrophilia and lymphocytosis. Blood smear examination showed intraerythrocytic inclusions morphologically consistent with small piroplasmids, and PCR amplification and sequencing of the *18S* rDNA gene confirmed infection with *B. banethi*. Screening for other vector‐borne pathogens scored negative. Treatment with imidocarb dipropionate resulted in temporary clinical and haematological improvement but persistence of PCR positivity and later relapse of parasitaemia and clinical signs. Subsequently, treatment with atovaquone/proguanil combined with azithromycin led to marked clinical recovery and disappearance of parasites at blood smear examination, although PCR positivity persisted for several months. A second therapeutic course resulted in both cytological and molecular negativity.

**Conclusions:**

This longitudinal case report provides the first detailed account of the clinical course, therapeutic response and long‐term parasitological follow‐up of *B. banethi* infection in a red fox, suggesting that this piroplasm might cause clinically relevant and persistent infection, yet further validation of this hypothesis on a larger number of clinical cases is advocated.

**Clinical study registration:**

Not applicable.

## Graphical abstract

A naturally occurring *Babesia banethi* infection in a sick red fox was followed up for more than one year through cytology, haematobiochemical profiles and molecular analyses. Imidocarb treatment resulted in temporary clinical improvement and later relapse, whereas atovaquone/proguanil combined with azithromycin led to clinical recovery and parasite clearance.

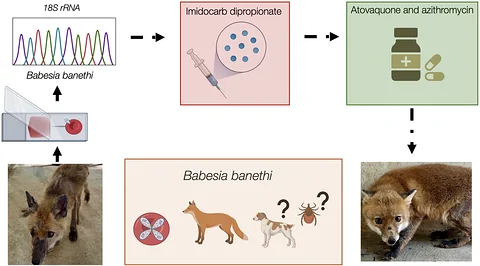

## 1 Introduction

Wildlife represents an important component in the ecology of tick populations as well as in the endemicity of tick‐borne pathogens (TBPs), mainly because of their condition of asymptomatic reservoirs for infections and the absence of antiparasitic preventative treatments (Otranto et al. 2015; Kazimírová et al. 2024). In this context, red foxes (*Vulpes vulpes*) serve as a key example of synanthropic carnivores widely distributed at a global scale, carrying both ticks and TBPs, including *Babesia* spp. (Birkenheuer et al. 2010; Cardoso et al. 2013; Baneth et al. 2019). *Babesia vulpes* is the dominant small piroplasm infecting foxes worldwide, with high molecular prevalence reported in several European countries (e.g., 42.6% in Italy and up to 70% in foxes from Spain and Portugal) (Baneth et al. 2019; Carbonara et al. 2026). In addition, this *Babesia* is of increasing veterinary relevance for disease in infected dogs, often causing anaemia, thrombocytopenia and azotaemia (Miró et al. 2015; Solano‐Gallego et al. 2016; Baneth et al. 2019).

On the other hand, the detection of a genetically distinct small *Babesia* species in foxes from Israel (Margalit Levi et al. 2018) and Iraq (Otranto et al. 2019) raised important questions regarding its life cycle and host specificity. The latter species was recently described as *Babesia banethi*, expanding the known span of species of piroplasmids infecting wild canids (Otranto et al. 2025). From the molecular and taxonomical perspective, phylogenetic analyses placed this new small *Babesia* species within clade III (i.e., Western group) of the piroplasmids, well apart from *B. vulpes* (clade I, *B*. *microti*‐like group) and from other *Babesia* species infecting domestic and wild carnivores (e.g., *Babesia canis*, *Babesia vogeli*, and *Babesia gibsoni* within the *Babesia* sensu stricto group, clade X) (Jalovecka et al. 2019).

Competent tick vectors of both *B. vulpes* and *B. banethi* have not been elucidated, although the parasite's DNA was detected in ticks collected from infected animals (e.g., *Ixodes hexagonus*, *I. ricinus*, *I. canisuga*, and *Rhipicephalus sanguineus* sensu lato for *B. vulpes*; *Ixodes kaiseri* for *B. banethi*) (Checa et al. 2018; Baneth et al. 2019; Otranto et al. 2025).

Unlike *B. vulpes*, for which increasing clinical data are available from infected dogs, information on *B. banethi* pathogenicity is currently limited (Carbonara et al. 2026) and lacking follow‐up data on its clinical course, response to treatment and parasitological cure in infected animals. This knowledge gap hampers the understanding of host‐parasite interactions and limits the development of evidence‐based therapeutic approaches, which is pivotal, in the context of wildlife rescue centres, to the reintroduction of animals into nature. This is important for ensuring the host animal's welfare while minimising the risk of *Babesia* transmission to other foxes and other canines.

Therefore, here we describe the clinical and parasitological course of a red fox naturally infected by *B. banethi*, providing the diagnostic protocol and therapeutic interventions in the absence of guidelines for managing babesiosis in wildlife.

## 2 Case Presentation

### 2.1 Initial Clinical Presentation and Diagnosis

In early August 2024, an adult male red fox was brought to the wildlife rescue centre in Bitetto (Apulia region, Southern Italy) due to severe debilitation. Specifically, the fox was rescued in Matera (Basilicata region) and transferred to the wildlife rescue centre, where its age class was determined according to Harris (1978). On physical examination (Video A, Supporting information), the animal was lethargic, dehydrated and underweight (i.e., 2.5 kg; the spinous processes of the vertebrae, ribs and pelvic bones were visible), with pale mucous membranes, brown discolouration of urine (e.g., suspected bloody urine) and presented extensive sarcoptic mange, confirmed by the observation of *Sarcoptes scabiei* mites in skin scrapes, but without evident ticks. Heart rate was 220 bpm, and the animal was tachypneic (i.e., higher than the physiological range of 21–40 breaths/min, Yatu et al. 2018).

**FIGURE 1:**
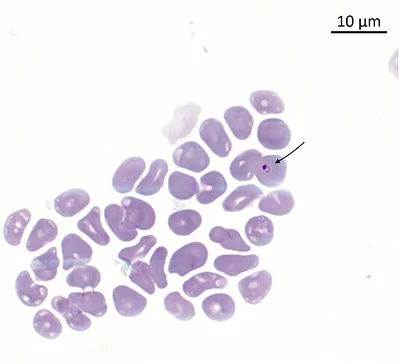
*Babesia banethi* merozoite (indicated by an arrow) retrieved in a fox blood smear, stained with Romanowsky solution, at 1000 × magnification.

Ivermectin (0.4 mg/kg, SC, 2 administrations every 14 days, for four weeks) and amoxicillin combined with clavulanic acid (7 mg/kg and 1.7 mg/kg respectively, BID, SC, for 10 days) were administered due to suspected urinary tract infection (the latter based on the brown colour of the urine) and sarcoptic mange. However, following this first course of drug administration, the animal clinically worsened, presenting more overt debilitation and paler mucous membranes. Only after one month from admission (September 2024; T‐1) was infection with a haemoparasite suspected and therefore a complete blood cell count (CBC) and biochemical profile were run, along with urinalysis and faecal examination (i.e., flotation and Baerman technique). While the coprology scored negative, the haematological evaluation (Gelli et al. 2023) (Table S1) revealed a regenerative anaemia with anisocytosis and polychromasia, Howell‐jolly bodies, rouleaux and increased reticulocytes; in association with leukocytosis characterised by neutrophilia with left shift and lymphocytosis. Abnormalities in serum biochemistry (Gelli et al. 2023) (Table S2) included increased alanine aminotransferase (ALT) and alkaline phosphatase (ALP) activities, as well as increased total bilirubin, reduced total proteins with hypoalbuminemia, increased cholesterol, amylase and lipase, and reduced Na/K ratio. Urinalysis showed the presence of haemoglobinuria/myoglobinuria at 50 µmol/L (value obtained from supernatant after centrifuging the urine sample) and haematuria (i.e., a medium level of blood and 20 red blood cells per high‐power field, 40X observation of urine sediment). Blood smear examination revealed the presence of intraerythrocytic inclusions morphologically consistent with small piroplasmids (Figure 1), prompting further molecular investigations for a species level identification. Deoxyribonucleic acid (DNA) was extracted from 200 µL of whole blood using a commercial kit (QIAamp DNA Blood Tissue, Qiagen, Hilden, and Germany) and was analysed by conventional PCR (cPCR) targeting the *18S* DNA gene (RLBF/R, ∼ 510 bp; Gubbels et al. 1999). The amplified PCR product was visualised by gel electrophoresis in a 2% agarose gel containing GelRed nucleic acid gel stain (VWR International PBI, Milan, Italy) and viewed on a ChemiDoc documentation system (BIORAD, Hercules, California and USA). The purified amplicon was sequenced and compared with those available in GenBank by the Basic Local Alignment Search Tool (BLAST‐http://blast.ncbi.nlm.nih.gov/Blast.cgi). The BLAST analysis revealed 99.6% nucleotide identity with the sequence of *B. banethi* (KJ956782). In addition, the animal was screened for antibodies reactive with *Leishmania infantum*, *Ehrlichia canis, Anaplasma phagocytophilum/platys*, and the presence of *Dirofilaria immitis* antigen, using the SNAP Leish 4Dx Test (IDEXX Laboratories, Westbrook, ME, USA) and for *Rickettsia* spp. DNA by cPCR (gltA gene, ∼400bp, Sgroi et al. 2021) and scored negative by all these tests. Therefore, based on the combination of clinical signs, haematological abnormalities and parasitological findings, a diagnosis of babesiosis was established, and subsequent therapeutic decisions were made according to the animal's clinical condition.

### 2.2 Therapeutic Protocol, Clinical and Parasitological Follow‐Up

**FIGURE 2:**
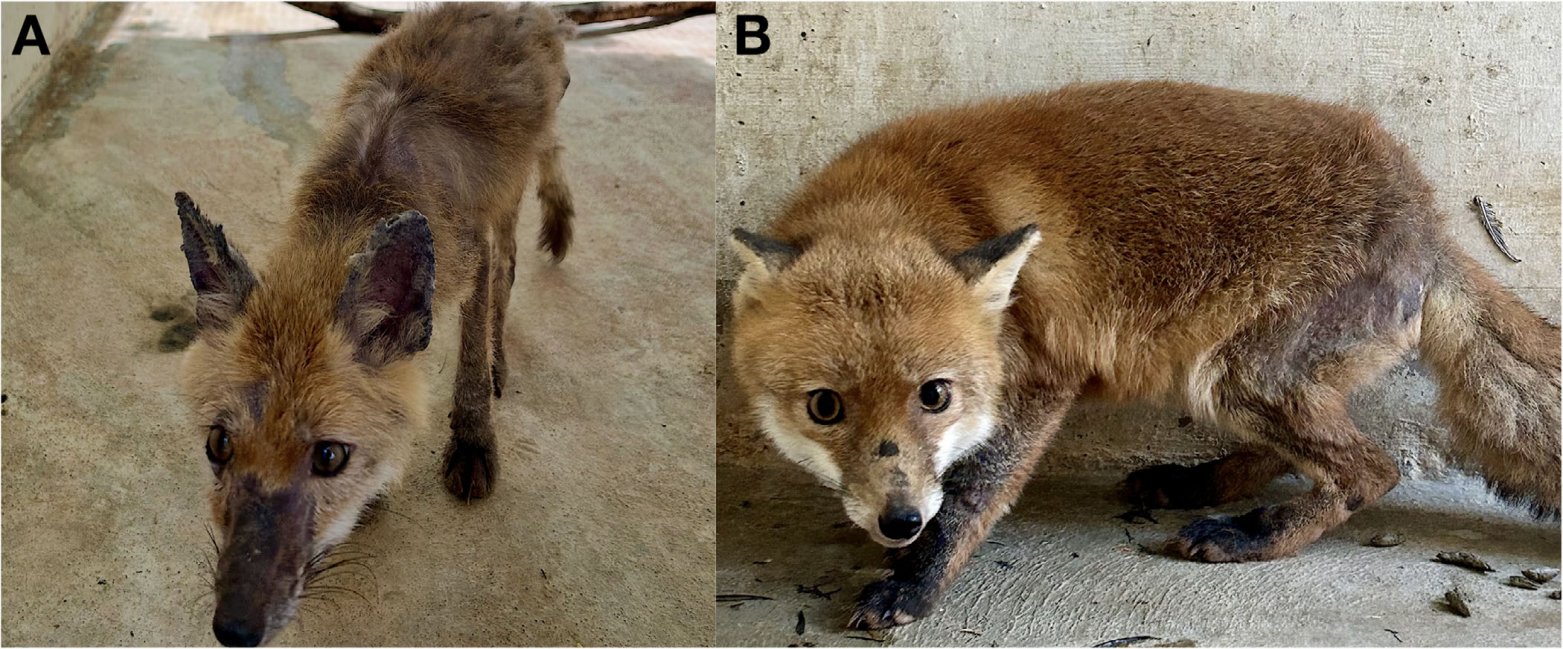
General presentation of a red fox with babesiosis due to *Babesia banethi* after the first dose of imidocarb dipropionate treatment (A) and after the first dose of atovaquone and proguanil hydrochloride in combination with azithromycin (B).

At the end of September 2024, the fox was treated with imidocarb dipropionate (T0), because it is the most commonly routinely used treatment for canine babesiosis (i.e., 6.6 mg/kg, SC, two doses at 28 days of distance) in the absence of guidelines for managing babesiosis in wildlife (Solano‐Gallego et al. 2016). Fifteen days after the first dose of imidocarb dipropionate (T1, +15) the fox showed clinical (Figure 2A, Video B, Supporting information) and haematological improvement, only with the persistence of lymphocytosis (Table S1), along with some serum biochemical alterations (Table S2). However, at T1, the parasitological follow‐up (i.e., blood smear observation and cPCR followed by sequencing, as described above) revealed a persistent *B. banethi* parasitaemia and PCR positivity. Two weeks after the second dose of imidocarb dipropionate (T2, +39), the fox continued to clinically improve; lymphocytosis and parasitaemia were not observed, but the animal was still positive for *B. banethi* DNA by PCR. After about three months from the second dose of the imidocarb (T3, +127, February 2025), the fox started to present again mild clinical signs of lethargy and pale mucous membranes, and laboratory alterations (i.e., anaemia, lymphocytosis and thrombocytosis) and parasitaemia recurred (Tables S1–2). The next follow‐up examinations (T4, +255 and T5, +281) revealed a consistent decline of the fox's health status, associated with the persistence of *B. banethi* infection (i.e., positivity at both blood smear and cPCR) (Tables S1–2). Therefore, almost one year after the initial diagnosis, a second therapeutic approach was started using atovaquone and proguanil hydrochloride (Malarone, GlaxoSmithKline, London, England) at 20 mg/kg, BID, OS and azithromycin (10 mg/kg, SID, SC), administered for 10 days following a general treatment regimen against small form canine *Babesia* spp. (Unterköfler et al. 2023; Antognoni et al. 2025). Again, a marked clinical and haematological improvement was observed after treatment (T6, +331 and T7, +352), which led to a negative blood smear but positive PCR for *B. banethi* persisted (Tables S1–2). About two months from the end of the Malarone therapy (T8, +379 and T9, +427) the fox was still parasitaemic but clinically stable (Figure 2B); however, several laboratory alternations, including lymphocytosis and hypergammaglobulinemia, were present. Hence, a SNAP Leish 4Dx Test was again performed but scored negative. In January 2026, the fox received a second course of the combination of Malarone and azithromycin, and one month from the end of this therapy (T10, +523) the animal was clinically stable (Video C, Supporting information), with persistent lymphocytosis and elevated liver enzymes but cytologically and molecularly negative for *B. banethi* (Tables S1–2).

## 3 Discussion

Data presented provide, for the first time, a longitudinal case follow‐up, describing the clinical course, therapeutic response and parasitological follow‐up of a natural *B. banethi* infection in a red fox from southern Italy. Specifically, the animal was a rescued fox, and all therapeutic decisions and follow‐up were based on its clinical condition, as in the case of the prolonged monitoring, which was due to the recurrence of clinical signs and persistence of parasitaemia, until clinical stabilisation and parasite negativity were achieved.

At presentation, the fox exhibited a clinical‐pathological picture consistent with initially acute and then chronic babesiosis (e.g., pale mucous membranes, weight loss, anaemia, and haemoglobinuria) (Solano‐Gallego et al. 2016). However, *Babesia* infection was not immediately investigated, as piroplasm infection in this animal species is generally asymptomatic (Baneth et al. 2019). For this reason, initial diagnostic and therapeutic procedures were orientated towards curing the most evident clinical findings (i.e., sarcoptic mange and brown urine). In this context, the severe sarcoptic mange and the poor nutritional status potentially prolonged the clinical course of the infection by *B. banethi* by impairing the host immune response. However, the absence of coinfections with other vector‐borne pathogens or gastrointestinal parasites, together with the recurrence of clinical signs and parasitaemia at light microscopy, corroborated the possibility for a primary pathogenic role for *B. banethi* in infected foxes.

Overall, the clinical, haematological and parasitological follow‐up showed a persistent PCR positivity associated with a fluctuating clinical status and parasitaemia, affected by drug administration and/or immunosuppression. Therefore, the prolonged intervals between follow‐up examinations were driven by the need to limit repeated handling of the animal; however, this approach may have delayed the timely detection of parasitaemia recurrence and, consequently, the administration of antiprotozoal treatment.

Imidocarb dipropionate was first administered because it is the most frequently used treatment for canine babesiosis in veterinary practice (Solano‐Gallego et al. 2016), requiring the animal prompt clinical intervention. In addition, no information was available on the clinical course and therapeutic approach of *B. banethi* infection. However, as expected from experience in canine medicine, this drug led to a transient clinical and haematological improvement, with a subsequent relapse of clinical signs and parasitaemia, thus confirming its limited efficacy against the small *Babesia* spp., as observed in dogs (Solano‐Gallego et al. 2016; Antognoni et al. 2025). Specifically, at T3, the recurrence of clinical signs was present but not severe, and the animal was still clinically stable. Therefore, considering that red foxes serve as asymptomatic reservoirs of piroplasmid infections (Baneth et al. 2019), close monitoring rather than immediate additional treatment was opted. Further treatment was implemented when the clinical condition worsened and parasitaemia persisted at the following examinations.

Accordingly, treatment with atovaquone/proguanil combined with azithromycin led to a marked and stable clinical‐haematological recovery, associated with negativity at blood smear examination, and, after the second dose, also at PCR. This pattern closely mirrors the course of small *Babesia* spp. infection in dogs, in which clinical remission does not correspond to rapid parasite clearance, with potential recrudescence events (Miró et al. 2015; Solano‐Gallego et al. 2016). Importantly, PCR positivity soon after drug administration should not be interpreted as treatment failure, as it might reflect the temporary persistence of the parasite's DNA rather than active babesiosis. For this reason, PCR testing has been recommended no earlier than two months after completion of treatment, particularly when monitoring small *Babesia* spp. (Solano‐Gallego et al. 2016). However, when PCR positivity persists for more than 3 months after treatment, the possible occurrence of the parasite's sequestration in target organs (e.g., spleen) and in circulating erythrocytes should be considered, as well as its potential reactivation following immunosuppressing events.

Overall, considering that rescued foxes should remain in rehabilitation facilities for as short a time as possible, the primary therapeutic efficacy indicator should be the clinical stabilisation and cytological negativity, rather than clearance of the parasite DNA. Finally, considering the off‐label use of atovaquone (Malarone), it is crucial to ensure therapeutic efficacy while limiting the risk of disseminating antimicrobial resistance, as recorded for *B. gibsoni* in dogs (Iguchi et al. 2020).

The slight thrombocytosis observed during the acute and relapse phases of the disease could reflect the rebound stimulation of the bone marrow in response to the haemolytic anaemia and differs from the thrombocytopenia commonly described in dogs infected with *B. vulpes* (Miró et al. 2015; Unterköfler et al. 2023). Conversely, most of the other haematological and biochemical abnormalities were consistent with those reported during small *Babesia* infections in dogs (e.g., anaemia, elevated liver enzymes, and increased total bilirubin) (Solano‐Gallego et al. 2016). However, fluctuations in leukocyte counts, particularly recurrent lymphocytosis, as well as hypergammaglobulinemia and hypoalbuminemia, are consistent with chronic immune stimulation also being reported in *B. vulpes* infections of dogs (Miró et al. 2015; Solano‐Gallego et al. 2016). Finally, the increased ALT and ALP activities, together with hyperbilirubinaemia and brown discolouration of urine, may reflect hepatocellular lesions secondary to haemolysis, anoxia and systemic inflammation, as reported in canine babesiosis (Solano‐Gallego et al. 2016). However, no specific hepatoprotective or additional supportive therapy was administered, which represents a limitation of this clinical management and should be considered when interpreting the persistence of liver enzyme alterations during follow‐up.

From a diagnostic perspective, this case highlights the complementary value of blood smear examination and molecular testing, which are of prime importance for identifying acute, chronic and relapse phases of the infection, with the latter important for species identification and for documenting the persistent infection status.

Overall, this longitudinal case report provides evidence that *B. banethi* might be a clinically relevant pathogen in red foxes by establishing persistent infection that is difficult to clear by medical treatment. In this context, considering that foxes are regarded as resistant to vector‐borne pathogens, data presented raise concerns about the clinical impact that *B. banethi* might have in more susceptible hosts, such as domestic dogs exposed to the infection by living in the same environment and being bitten by potentially vector ticks.

However, all the clinical assumptions above need further validation on a larger number of babesiosis cases due to *B. banethi*.

The limitations of this study include the delayed performance of haematological tests, blood smear and molecular examinations, as well as the absence of additional supportive therapy. Similarly, the earlier administration of atovaquone/proguanil hydrochloride and azithromycin, as a first line protocol, could have shortened the course of disease by providing timely cytological and then also molecular clearance of the parasite.

## Author Contributions

**Mariaelisa Carbonara**: conceptualisation, data curation, investigation, formal analysis, methodology, writing – original draft, writing – review and editing. **Michela Prioletti**: data curation, investigation, methodology, writing – review and editing. **Antonio Camarda**: data curation, methodology, writing – review and editing. **Stefano Castellana**: methodology, writing – review and editing. **Giuseppe Menga**: methodology, writing – review and editing. **Giovanna Girardi**: methodology, writing – review and editing. **Gad Baneth**: data curation, investigation, methodology, writing – review and editing. Domenico Otranto: conceptualisation, investigation, supervision, writing – review and editing.

## Funding

The authors have nothing to report.

## Ethics Statement

The fox was handled with regard to its well‐being, and the protocol for this study was approved by the ethical committee of the University of Bari, Italy (authorization number 337402).

## Conflicts of Interest

The authors declare no conflicts of interest.

## Supporting information




**Supporting File 1**: vms371184‐sup‐0001‐SuppMat.xlsx

**Supporting File 2**: vms371184‐sup‐0002‐SuppMat.docx

**Supporting File 3**: vms371184‐sup‐0003‐SuppMat.mov

**Supporting File 4**: vms371184‐sup‐0004‐SuppMat.mov

**Supporting File 5**: vms371184‐sup‐0005‐SuppMat.mp4

**Supporting File 6**: vms371184‐sup‐0006‐SuppMat.mp4

**Supporting File 7**: vms371184‐sup‐0007‐SuppMat.mov

**Supporting File 8**: vms371184‐sup‐0008‐SuppMat.docx

## Data Availability

All data supporting the main conclusions of this study are included in the manuscript. Raw data are available from the corresponding author upon reasonable request. No datasets were generated or analysed during the current study.
