# Supplementary material for: Clinical Case and Follow‐Up of Babesia banethi Infection in a Red Fox
Source: Vet Med Sci. 2026 Aug 27;12(5):e71184. doi: 10.1002/vms3.71184 (PMC13519672; doi:10.1002/vms3.71184)
Supplement: Supplementary file 1 [file VMS3-12-e71184-s001.docx]

**Supporting information Video A**: General presentation of a red fox with sarcoptic mange and babesiosis due to *Babesia banethi*. First clinical assessment, before drug administration.

**Supporting information Video B**: General presentation of a red fox with babesiosis due to *Babesia banethi*. Clinical assessment performed after the first dose of imidocarb dipropionate treatment.

**Supporting information Video C**: General presentation of a red fox cured from babesiosis due to *Babesia banethi*. Clinical assessment performed after the second dose of atovaquone and proguanil hydrochloride in combination with azithromycin.
