# Supplementary material for: Clinical Case and Follow‐Up of Babesia banethi Infection in a Red Fox
Source: Vet Med Sci. 2026 Aug 27;12(5):e71184. doi: 10.1002/vms3.71184 (PMC13519672; doi:10.1002/vms3.71184)
Supplement: Supplementary file 2 [file VMS3-12-e71184-s003.docx]

**Figure captions:**

**Figure 1.** *Babesia banethi* merozoite (indicated by an arrow) retrieved in fox blood smear, stained with Romanowsky solution, at 1000 × magnification.

**Figure 2.** General presentation of a red fox with babesiosis due to *Babesia banethi* after the first dose of imidocarb dipropionate treatment (A) and after the first dose of atovaquone and proguanil hydrochloride in combination with azithromycin (B).
