# Supplementary material for: Clinical Case and Follow‐Up of Babesia banethi Infection in a Red Fox
Source: Vet Med Sci. 2026 Aug 27;12(5):e71184. doi: 10.1002/vms3.71184 (PMC13519672; doi:10.1002/vms3.71184)
Supplement: Supplementary file 3 [file VMS3-12-e71184-s005.docx]

A naturally occurring Babesia banethi infection in a sick red fox was followed-up for more than one year, through cytology, hematobiochemical profiles and molecular analyses. Imidocarb treatment resulted in temporary clinical improvement and later relapse, whereas atovaquone/proguanil combined with azithromycin led to clinical recovery and parasite clearance.
