# Supplementary material for: Clinical Case and Follow‐Up of Babesia banethi Infection in a Red Fox
Source: Vet Med Sci. 2026 Aug 27;12(5):e71184. doi: 10.1002/vms3.71184 (PMC13519672; doi:10.1002/vms3.71184)
Supplement: Supplementary file 4 [file VMS3-12-e71184-s007.docx]

**Supplementary Table 1.** Longitudinal hematological findings recorded in alive red fox infected with *Babesia banethi,* in relation to diagnostic test and medical treatments.

**Supplementary Table 2.** Longitudinal biochemical findings recorded in alive red fox infected with *Babesia banethi,* in relation to diagnostic test and medical treatments.
